# Multidisciplinary oral rehabilitation in partially edentulous adult patients with malocclusion: A cross-sectional survey study

**DOI:** 10.4317/jced.55282

**Published:** 2018-12-01

**Authors:** Renan Devita, Sérgio Pinho, Josep-Maria Ustrell, Henrique Pretti, Esdras-de Campos França, Ertty Silva, Igor Brum

**Affiliations:** 1 Rua do Ouvidor 183, sala 307, Centro, Rio de Janeiro, RJ, Brazil; 2 Residencial Jardins do Lago Quadra 09, Rua Bouganville casa 04, Bairro Jardim Botȃnico, Brasilia, Brazil; 3 Full Professor, Vice-Dean of Odontology, Faculty of Medicine and Health Sciences, University of Barcelona, Group of Oral Health and Masticatory System, Instituto de Investigación Biomédica de Bellvitge (IDIBELL), L’Hospitalet de Llobregat, Barcelona, Spain; 4 Full Professor, Director of the Faculty of Dentistry of the Federal University of Minas Gerais (UFMG). Av. Antonio Carlos 6627, Campus Pampulha, Belo Horizonte, Brazil; 5 Avenida do Contorno 4480, sala 1104, Funcionàrios, Belo Horizonte, Brazil; 6 SHIS Q1 07 Bloco C 104, Lago Sul, Brasilia, Brazil; 7 Rua Aurelino Leal 14, Centro, Niterói, RJ, Brazil

## Abstract

**Background:**

A cross-sectional survey was conducted to gather information regarding the opinion of Brazilian specialists in both orthodontics and implantology on multidisciplinary oral rehabilitation in partially edentulous patients with malocclusion.

**Material and Methods:**

A total of 305 specialists participated in a telephone survey and answered an ad hoc 10-item questionnaire, including the request of total skull cone-beam tomographies (CBCT) and the use of 3D digital planning software, the best moment of treatment to place dental implants, and the integration of orthodontics in implantology.

**Results:**

Most participants did not request CBCT (90.8%) or 3D digital planning software images (92.3%) to diagnose and plan multidisciplinary oral rehabilitation. By contrast, 91.1% of participants would use an already dental implant as anchorage for orthodontics, 73.8% had already used implants for this purpose, 47.9% selected 4 months as the waiting time between implant placement and its use as anchorage, and 58.4% had already placed dental implants having in mind using them as anchorage for orthodontics and anticipating the oral rehabilitation process. Moreover, 93.4% of participants stated to avoid applying orthodontic forces in implants with unfavourable prognosis. A total of 67.9% of participants got the degree of specialist in Orthodontics before that of specialist in Implantology. The main reason for obtaining the other specialty degree was to be able to thoroughly exercise the two specialties.

**Conclusions:**

The use of technological advances, such as CBCT and 3D digital planning software was limited. Most dental specialists would wait the osseointegration recommended time before applying orthodontic forces and thus using them as anchorage for orthodontics. The majority of interviewed dentists sought the other specialty to acquire multidisciplinary knowledge.

** Key words:**Cross-sectional study, orthodontics, implantology, partially edentulous, malocclusion, oral rehabilitation.

## Introduction

Occlusal rehabilitation of partially edentulous adult patients who do not want a removable partial denture continues to be a challenge for dental specialists in routine daily practice. The use of dental implants-supported fixed prosthesis has shown to offer benefits over a tooth-soft tissue supported removable partial denture prosthesis (1). However, a comprehensive evaluation, multidisciplinary approach and a sequential treatment plan worked out in harmony with the patient’s perceptions are important for a long-term successful outcome. After establishing the diagnosis, it is important to determine the best moment for carrying out the orthodontic treatment. On the other hand, the use of implants for orthodontic anchorage requires an interdisciplinary approach and precise planning to achieve optimal results (2,3). It is recommended that placement of dental implants should be done after completion of orthodontic treatment, although immediate loading of rough-surfaced, screw-type implants supporting fixed dentures for the treatment of edentulous maxilla or mandible appears to be a reliable treatment option with a high probability of success (4). However, in partially edentulous patients with malocclusion the ideal time of implantation has not been clearly established. Successful results have been reported in patients with class II and class III malocclusion using min implants anchorage (5,6).

The crucial role of correct diagnosis and careful planning of treatment approach based on individual characteristics of the patients has become widely recognized. In this respect, total skull cone-beam tomography (CBCT) has proven its value in dental practice when conducting craniofacial measurements for the 3D visualization of the craniofacial complex from different perspectives (7). Different studies have shown the superiority of CBCT as compared to conventional cephalometric images for assessing malocclusion and asymmetry (8,9). In addition, in order to define the most suitable treatment plan, there must be a dental specialist team working synchronically for defining the most suitable treatment plan to accomplish stable occlusion and facial harmony. If a single dentist intends to conduct a multidisciplinary oral rehabilitation for a partially edentulous patient with malocclusion, expertise in different specialties for diagnosing, planning, and performing treatment is necessary.

Therefore, the current study aimed to collect information regarding the opinion of dental specialists in both orthodontics and implantology on the multidisciplinary oral rehabilitation approach for partially edentulous adult patients with malocclusion.

## Material and Methods

-Study design

A cross-sectional survey was conducted in Brazil between January and May 2018. The objective of the study was to gather information on the following aspects related to oral rehabilitation of adult patients with malocclusion and partially edentulous arches: a) the use of CBCT and 3D digital planning software; b) the use of osseointegrated dental implants as anchorage for orthodontic treatment; c) the best moment of the orthodontic treatment for placing dental implants; and d) the integration of implantology in the orthodontic treatment of partially edentulous patients with malocclusion.

The study was approved by the Brazilian Ethics Committee (Plataforma Brasil; registration CAAE 56757116.7.0000.5646 and opinion number 1.644.556). The Brazilian Federal Council of Dentistry (Conselho Federal de Odontologia, CFO) granted, by signing a confidentiality agreement with the principal investigator (R.D.) who was responsible for the study, the telephone contact list of all dentist who were duly registered as specialists in both orthodontics and implantology at the year 2017.

-Participants

The sample size was defined by the total number of 507 specialists in both orthodontics and implantology registered at CFO database in 2017. Inclusion criteria were as follows: 1) to have available an updated telephone contact number in the CFO database, 2) to be available to answer the telephone call, and 3) to provide oral consent to participate in the study with an affirmative answer to this introductory explanation at the beginning of the telephone call: “Dear colleague, like you, I am a specialist in orthodontics and implantology. I am doing a doctorate study and this research was approved by Plataforma Brasil (Ethics Committee). We got your phone contact through the Brazilian Federal Council of Dentistry (CFO). Do you agree to participate in this study and answer a 10-item questionnaire on multidisciplinary oral rehabilitation for partially edentulous adult patients with malocclusion?” The study included the possibility of scheduling for the best time for the participant. For each name in the CFO list, up to three phone calls on different days and times to try to reach the participant were made.

-Study questionnaire

The study questionnaire was designed ad hoc by the principal investigator (R.D.) who is a dental specialist in orthodontics and implantology and a student to obtain the degree of Doctorate in Dentistry. The suitability of the questionnaire was reviewed by one of the senior authors (J.M.U.). As shown in  Table 1, the questionnaire had 10 multiple-choice simple questions most of them with a “yes” or “no” categorical answer. Demographic questions were not included in the survey and responses were anonymized. The expected time to complete the interview should take no longer than 3 minutes, but participants were able to request additional explanations.

**Table 1:**
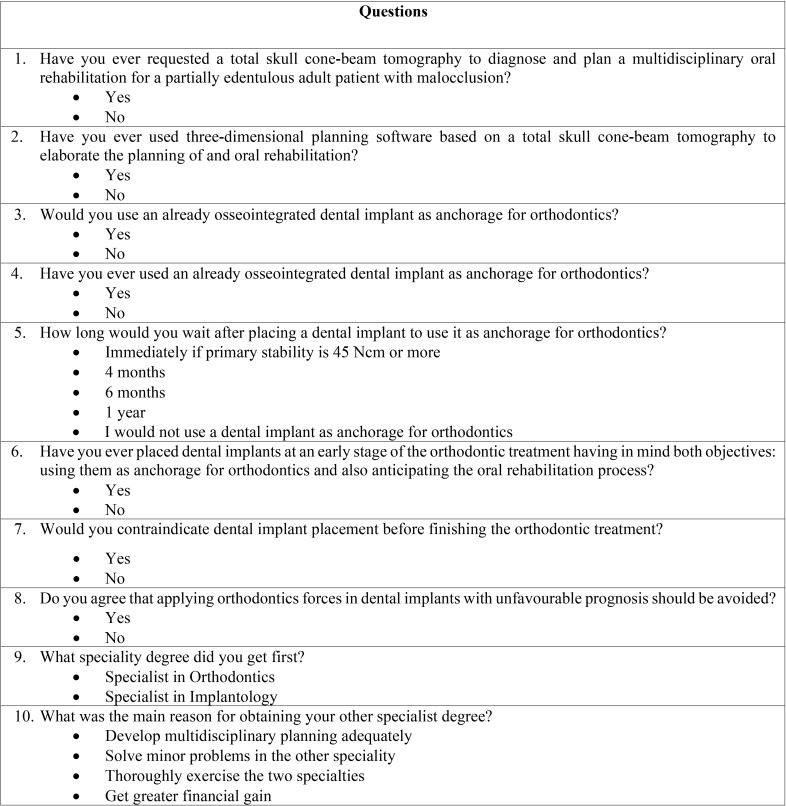
Details of the study questionnaire.

-Statistical analysis

Of a total of 507 eligible specialists in orthodontics and implantology, a simple random sampling method without replacement was used. A 95% confidence interval and a margin error of 4 percentage points were defined, which resulted in a sample size of 305 participants with a margin error of 3.6 percentage points. Responses were computed using the SurveyMonkey® platform (https://www.surveymonkey.com). Data are expressed as frequencies and percentages. The chi-square (χ2) test or the Fisher’s exact test was used for the comparison of categorical variables. Statistical significance was set at *P* < 0.05.The SPSS statistical package 24.0 (SPSS, Chicago, IL) was used for the analysis of data.

## Results

**Figure 1:**
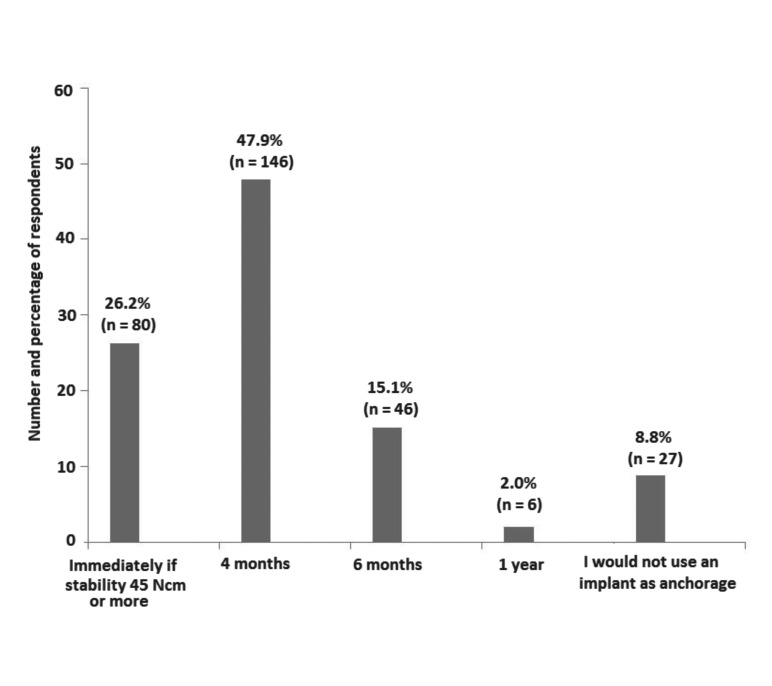
Responses to the question of how long would you wait after placing a dental implant to use it as anchorage for orthodontics?

Answers to the study questionnaire are shown in  Table 2. In relation to the request of CBCT and the use of 3D digital planning software to diagnose and plan a multidisciplinary oral rehabilitation for partially edentulous patients with malocclusion, most participants gave a negative answer (90.8% and 92.3%, respectively). By contrast, 91.1% of participants would use an already dental implant as anchorage for orthodontics and 73.8% had already used implants as anchorage for orthodontic treatment. Also, almost half of participants (47.9%) selected 4 months as the waiting time between implant placement and its use as anchorage (Fig. 1).

**Table 2:**
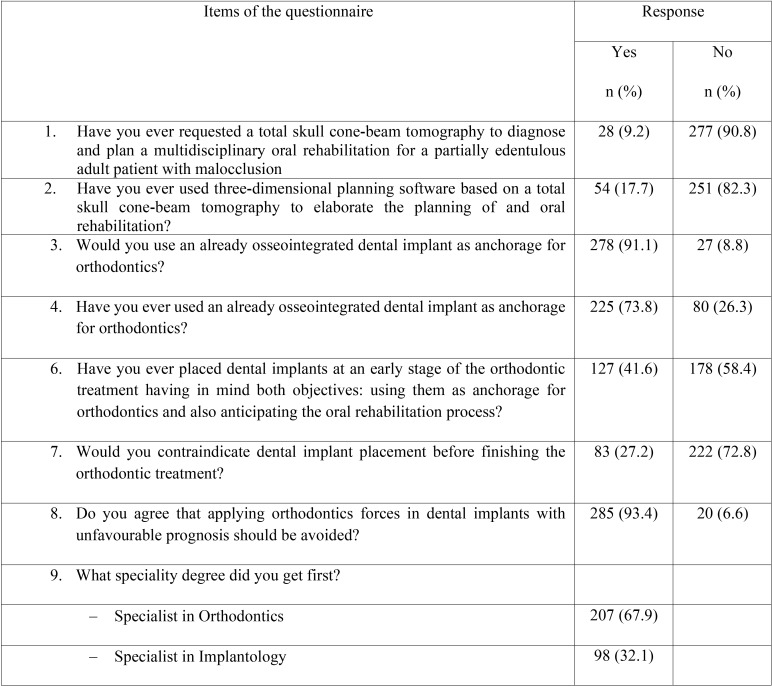
Answers to the study questionnaire of 305 participants.

More than half of participants (58.4%) had already placed dental implants having in mind using them as anchorage for orthodontics and anticipating the oral rehabilitation process. Moreover, 93.4% of participants stated to avoid applying orthodontic forces in implants with unfavourable prognosis ( Table 2).

A total of 67.9% (n = 207) of participants got the degree of specialist in Orthodontics before that of specialist in Implantology. Regarding the main reason for obtaining the other specialty degree, 42.9% (n = 131) considered to be able to thoroughly exercise the two specialties, 24.6% (n = 75) to develop multidisciplinary planning adequately, 19% (n = 58) to get greater financial gain, and 13.4% (n = 41) to solve minor problems in the other specialty. As shown in  Table 3, the percentages of specialists who obtained the second degree either in Implantology or Orthodontics was similar for the reasons of “thoroughly exercise in the two specialties” (42.5% and 43.9%) and “to solve minor problems in the other specialty” (14.5% and 11.2%). However, a higher percentage of specialists in Implantology first obtained the second degree in Orthodontics “to develop multidisciplinary planning adequately” (34.7% vs 19.8%), whereas a higher percentage of specialists in Orthodontics first obtained the second degree in Implantology “to get greater financial gain” (23.2% vs 10.2%). In both cases, differences were significant (*P* = 0.006).

**Table 3:**
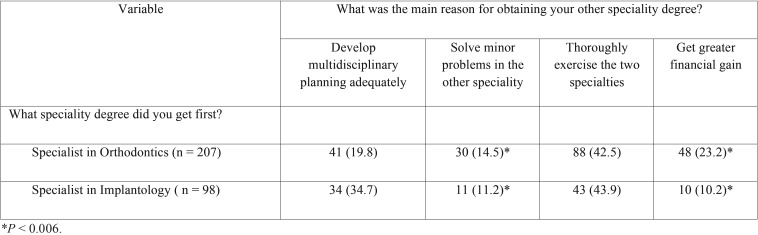
Reasons for obtaining the second degree in Implantology or Orthodontics.

There was a significant relationship between the use of osseointegrated dental implant as anchorage and requesting CBCT, that is, a higher percentage of participants who used a dental implant for anchorage requested CBCT scanning to diagnose and plan multidisciplinary oral rehabilitation as compared to those who had never used a dental implant as anchorage (11.1% [25/225] vs. 3.7% [3/80], *P* < 0.05).

## Discussion

In order to obtain high predictability in the treatment of complex cases, such as multidisciplinary oral rehabilitations in partially edentulous adult patients with malocclusion, it was expected that a CBCT would have been widely requested by dental specialists in both orthodontics and implantology. This diagnostic modality provides 3D images of the entire craniofacial anatomical region, which seems of great help to establish an accurate diagnosis (10-12). The cost-benefits of CBCT scanning are superior to the combination of several 2-dimensional (2D) radiographic images with respect to the intrinsic information, and to CT with respect to radiation dose and cost. The replacement of conventional plain radiographs with the 3D-capable devices appears to be an unavoidable current trend (13). However, an unexpected finding of the survey was the very low percentage of participants who were familiar with these imaging techniques (9.2% and 17.7% with CBCT and 3D planning software, respectively), and the significant association between the use of dental implants as anchorage and requesting CBCT scans.

The small percentage of participants who were familiar with CBCT and 3D3D digital planning software is still more striking considering that specialists in both orthodontics and implantology was an inclusion criteria of the study. The survey was not designed to assess the reasons behind answers to the questionnaire, but at least our results provide evidence of the low penetration of CBCT technology in Brazilian dental practice, particularly for assessing and planning oral rehabilitation in partially edentulous adult patients with associated malocclusion.

Although diverse treatment approaches involving different sequence procedures 

can be used in the management of partially edentulous patients with malocclusion (14-16), protocols combining orthodontics and implant therapy have shown successful functional and esthetic results as well as improvement of quality of life and self-esteem (17,18). In fact, implants are commonly used to replace missing teeth in partially edentulous adult orthodontic patients. Because these patients are missing teeth, orthodontic mechanics may be complicated or often impossible because of insufficient anchorage. In these situations even aggravated with the presence of malocclusion, it may be feasible to use the implant initially as an orthodontic anchor to facilitate complex tooth movement and secondarily as an abutment for a crown or fixed prosthesis (19).

In agreement with the evidence, 91.1% of participants would use an already osseointegrated dental implant as anchorage for orthodontic treatment, although 73.8% of them reported to have had experience with the use of implants as anchorage. Also, 72.8% would contraindicate placement of implants before finishing the orthodontic treatment. The majority of participants (93.4%) agreed that applying orthodontics forces in dental implants with unfavourable prognosis should be avoided.

Regarding the waiting time required for the use of a dental implant as anchorage for orthodontics, recommendations in the literature can vary from the immediate loading with orthodontic forces soon after provisional prosthesis confection or the loading only after the osseointegration (20-22). Factors such as the bone quality of the implant site (bone type), the macro design of the dental implant, the primary stability and also the quality of the surface treatment of the implants are essential for individualized treatment planning (16,23,24). Almost half of participants (47.9%) selected 4 months as the ideal waiting time, although 26.2% reported placement of the implant immediately with orthodontic forces when necessary, if primary stability is ≥ 45 Ncm. However, timing of implant placement in partially edentulous patients with maloclusión should be defined case by case on the basis of multidisciplinary treatment planning as there is insufficient evidence to determine the possible advantages or disadvantages of immediate, immediate-delayed or delayed implants (25).

In order to perform adequately multidisciplinary oral rehabilitation in partially edentulous patients with malocclusion, it is necessary to acquire skills in both orthodontic procedures and implant therapy (26,27). Most of participants were specialists in Orthodontics first. Regarding the reasons to get the second specialty degree, a similar percentage of participants stated “thoroughly exercise the two specialties” and “to solve minor problems in the other specialty”. However, it appears that a higher percentage of specialists in Orthodontics first pursued the second degree in Implantology to get greater financial gain. Interestingly, a higher percentage of specialists in Implantology first obtained the second degree in Orthodontics to develop multidisciplinary planning adequately.

The present results should be interpreted taking into account limitations of the study, including the lack of information regarding the number of telephone calls made to reach participants or duration of the interviews. We used an ad hoc questionnaire, which has been shown to be an inexpensive tool, highly accepted from patients and reliable tool recommended to expedite systematic collection of relevant clinical data in different settings (28,29). The instrument however, has not been validated and was originally developed in Portuguese. Also, the study participants were Brazilian specialists in Orthodontics and Implantology, which may limit generalizability of results to other populations of dentists. However, the information collected is clinically relevant and provides evidence of the current status of dental practice in the multidisciplinary oral rehabilitation for partially edentulous patients with malocclusion.

## References

[1] Madani AS, Moeintaghavi A, Rezaeei M (2010). Occlusal rehabilitation in a partially edentulous patient with lost vertical dimension using dental implants: a clinical report. J Contemp Dent Pract.

[2] Rose TP, Jivraj S, Chee W (2006). The role of orthodontics in implant dentistry. Br J Dent.

[3] Papadopoulos MA, Tarawneh F (2007). The use of miniscrew implants for temporary skeletal anchorage in orthodontics: a comprehensive review. Oral Surg Oral Med Oral Pathol Oral Radiol Endod.

[4] Strietzel FP, Karmon B, Lorean A, Fischer PP (2011). Implant-prosthetic rehabilitation of the edentulous maxilla and mandible with immediately loaded implants: preliminary data from a retrospective study, considering time of implantation. Int J Oral Maxillofac Implants.

[5] Baek SH, Yang IH, Kim KW, Ahn HW (2011). Treatment of class iii malocclusions using miniplate and mini-implant Anchorage. Semin Orthod.

[6] de Lima E, Brum F, Mezomo M, et al (2017). Orthodontic treatment of Class III malocclusion with lower extraction and anchorage with mini implants: Case report. J World Fed Orthod.

[7] Kapila SD, Nervina JM (2015). CBCT in orthodontics: assessment of treatment outcomes and indications for its use. Dentomaxillofac Radiol.

[8] Minich CM, Araújo EA, Behrents RG,  Buschang PH,  Tanaka OM,  Kim KB (2013). Evaluation of skeletal and dental asymmetries in Angle Class II subdivision malocclusions with cone-beam computed tomography. Am J Orthod Dentofacial Orthop.

[9] Huang M, Hu Y, Yu J, Sun J,  Ming Y,  Zheng L (2017). Cone-beam computed tomographic evaluation of the temporomandibular joint and dental characteristics of patients with Class II subdivision malocclusion and asymmetry. Korean J Orthodond.

[10] Agrawal JM, Agrawal MS, Nanjannawar LG, Parushetti AD (2013). CBCT in orthodontics: the wave of future. J Contemp Dent Pract.

[11] American Academy of Oral and Maxillofacial Radiology (2013). Clinical recommendations regarding use of cone beam computed tomography in orthodontics. [corrected]: position statement by the American Academy of Oral and Maxillofacial Radiology. Oral Surg Oral Med Oral Pathol Oral Radiol.

[12] Berco M, Rigali PH, Miner RM, DeLuca S, Anderson NK, Will LA (2009). Accuracy and reliability of linear cephalometric measurements from cone-beam computed tomography scans of a dry human skull. Am J Orthod Dentofacial Orthop.

[13] Cattaneo PM, Bloch CB, Calmar D, Hjortshøj M, Melsen B (2008). Comparison between conventional and cone-beam computed tomography-generated cephalograms. Am J Orthod Dentofacial Orthop.

[14] Blanco Carrión J, Ramos Barbosa I, Pérez López J (2009). Osseointegrated implants as orthodontic anchorage and restorative abutments in the treatment of partially edentulous adult patients. Int J Periodontics Restorative Dent.

[15] Bidra AS, Uribe F (2012). Preprosthetic orthodontic intervention for management of a partially edentulous patient with generalized wear and malocclusion. J Esthet Restor Dent.

[16] Roberts WE, Engen DW, Schneider PM, Hohlt WF (2004). Implant-anchored orthodontics for partially edentulous malocclusions in children and adults. Am J Orthod Dentofacial Orthop.

[17] Huang LH, Shotwell JL, Wang HL (2005). Dental implants for orthodontic anchorage. Am J Orthod Denetofacial Orthop.

[18] Johal A, Alyaqoobi I, Patel R, Cox S (2015). The impact of orthodontic treatment on quality of life and self-esteem in adult patients. Eur J Orthodont.

[19] Kokich VG (1996). Managing complex orthodontic problems: the use of implants for anchorage. Semin Orthod.

[20] Mangano C, Raes F, Lenzi C, Eccellente T, Ortolani M, Luongo G (2017). Immediate loading of single implants: A 2-year prospective multicenter study. Int J Periodontics Restorative Dent.

[21] Lanza A, Di Francesco F, De Marco G, Scognamiglio F1 Aruta V, Itro A (2017). Multidisciplinary approach in the management of a complex case: Implant-prosthetic rehabilitation of a periodontal smoking patient with partial edentulism, malocclusion, and aesthetic diseases. Case Rep Dent.

[22] Budtz-Jörgensen E (1996). Restoration of the partially edentulous mouth--a comparison of overdentures, removable partial dentures, fixed partial dentures and implant treatment. J Dent.

[23] Davarpanah K, Decker A, Sache MP, Deffrennes D, Demurashvili G, Szmukler-Moncler S (2014). New protocol combining orthodontics and implant therapy for partially edentulous adult patients. Part I: Description of the Decker protocol. [Article in French]. Rev Stomatol Chir Maxillofac Chir Orale.

[24] Moy PK, Medina D, Shetty V, Aghaloo TL (2005). Dental implant failure rates and associated risk factors. Int J Oral Maxillofac Implants.

[25] Esposito M, Grusovin MG, Polyzos IP, Felice P, Worthington HV (2010). Timing of implant placement after tooth extraction: immediate, immediate-delayed or delayed implants? A Cochrane systematic review. Eur J Oral Implantol.

[26] Rose TP, Jivraj S, Chee W (2006). The role of orthodontics in implant dentistry. Br Dent J.

[27] Chiu G, Chang C, Roberts WE (2018). Interdisciplinary treatment for a compensated Class II partially edentulous malocclusion: Orthodontic creation of a posterior implant site. Am J Orthod Dentofacial Orthop.

[28] Guaraldi F, Gori D, Beccuti G, Prencipe N, Giordano R, Mints Y (2016). Usefulness of an ad hoc questionnaire (Acro-CQ) for the systematic assessment of acromegaly comorbidities at diagnosis and their management at follow-up. J Endocrinol Invest.

[29] Cinti ME, Cannavò M, Fioravanti M (2017). Stress at work: development of the Stress Perception Questionnaire of Rome (SPQR), an ad hoc questionnaire for multidimensional assessment of work related stress. Clin Ther.

